# The hydrodynamics and kinematics of the appendicularian tail underpin peristaltic pumping

**DOI:** 10.1098/rsif.2023.0404

**Published:** 2023-11-15

**Authors:** Terra C. Hiebert, Brad J. Gemmell, George von Dassow, Keats R. Conley, Kelly R. Sutherland

**Affiliations:** ^1^ Oregon Institute of Marine Biology, University of Oregon, OR 97420, USA; ^2^ Department of Integrative Biology, University of South Florida, Tampa, FL, USA

**Keywords:** *Oikopleura dioica*, temperature, fluid dynamics, filter feeding, plankton, peristaltic pump

## Abstract

Planktonic organisms feed while suspended in water using various hydrodynamic pumping strategies. Appendicularians are a unique group of plankton that use their tail to pump water over mucous mesh filters to concentrate food particles. As ubiquitous and often abundant members of planktonic ecosystems, they play a major role in oceanic food webs. Yet, we lack a complete understanding of the fluid flow that underpins their filtration. Using high-speed, high-resolution video and micro particle image velocimetry, we describe the kinematics and hydrodynamics of the tail in *Oikopleura dioica* in filtering and free-swimming postures. We show that sinusoidal waves of the tail generate peristaltic pumping within the tail chamber with fluid moving parallel to the tail when filtering. We find that the tail contacts attachment points along the tail chamber during each beat cycle, serving to seal the tail chamber and drive pumping. When we tested how the pump performs across environmentally relevant temperatures, we found that the amplitude of the tail was invariant but tail beat frequency increased threefold across three temperature treatments (5°C, 15°C and 25°C). Investigation into this unique pumping mechanism gives insight into the ecological success of appendicularians and provides inspiration for novel pump designs.

## 1. Background

Pumps in nature generate flow by adding energy to fluid [1,2] and can enable both internal fluid movement in a static organism or can generate whole-organism locomotion. Planktonic organisms exhibit a diversity of pumping strategies [3–5] for locomotion and feeding while suspended in the water column. Pelagic tunicates (phylum: Chordata, subphylum: Tunicata) are planktonic filter feeders that concentrate suspended food particles by pumping water and prey particles across fine mucous meshes. Their effective propulsion and high filtering efficiency on minute particles, from microzooplankton down to viruses [6–10], have inspired design solutions (e.g. [11–15]).

**Figure 1.**
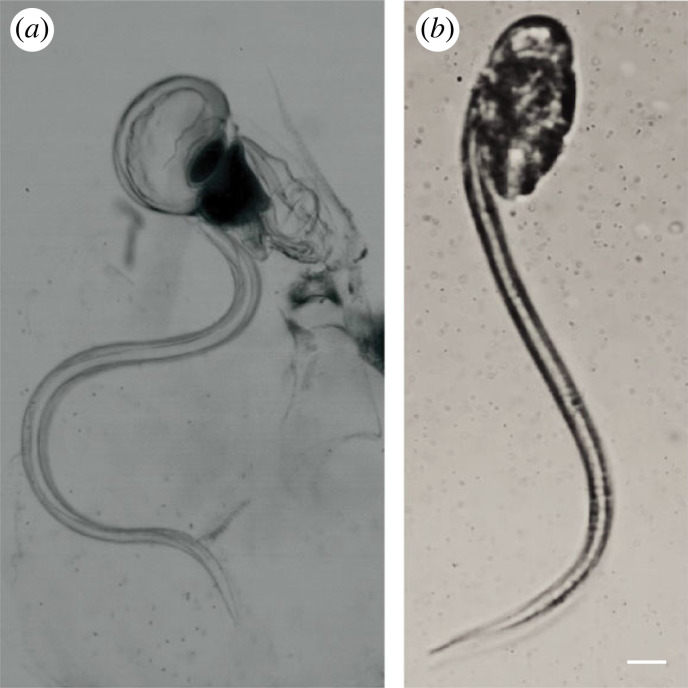
Comparison of appendicularian tail wave characteristics when filtering (*a*) and free-swimming (*b*). Note differences in postures including tail wave shape and amplitude, and trunk orientation. Scale bar 100 µm.

All pelagic tunicates filter small particles from the water using mucous mesh filters, but how flow is driven across filters differs among taxa [10]. Appendicularians (family: Appendicularia or Larvacea) generate flow with a tadpole-like tail that is attached to their body or trunk [10,16,17]. Appendicularian swimming and feeding occurs by rapid tail beating, originating from two muscle bands that alternatively contract along its length [18,19], but the associated tail wave characteristics differ (figure 1) [19]. When feeding, the appendicularian remains relatively static and the tail drives water over mucous mesh filters.

**Figure 2.**
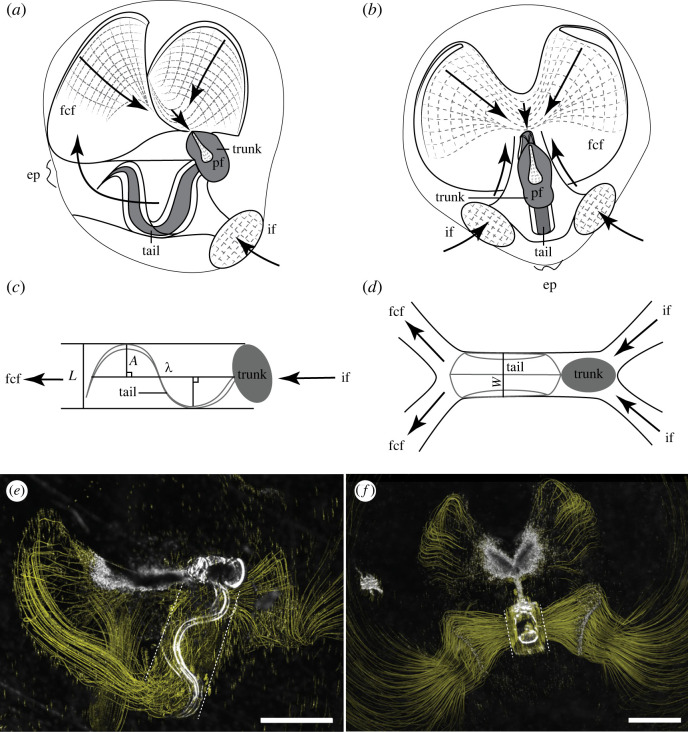
Flow through the appendicularian house is driven by tail beating. Schematic of house morphology in side (*a*) and dorsal (*b*) views. The tail is connected to the trunk and situated within a tail chamber. Fluid with suspended food particles is drawn into the house through two inlet filters (if), which coarsely filter large particles. Flow moves through the tail chamber and through two food concentrating filters (fcf). Concentrated particles on the fcf then move to a pharyngeal filter (pf) before being ingested. Excluded particles are expelled through an exit port (ep). (For a full description of *Oikopleura dioica* house morphology see fig. 1 in [20]). Simplified schematics of tail chamber are shown in side (*c*) and top views (*d*); appendicularian trunk is grey oval and fluid movement is shown with black arrows. Morphological and tail wave characters are shown and include tail chamber width *L* (*c*), tail width *W* (*d*), and wave amplitude *A* (*c*) and wavelength λ (*c*). The tail contacts the tail chamber walls with wave amplitude and tail width defined by the tail chamber dimensions. Particle paths through the house are visualized from a stack of superimposed images in lateral (*e*) and dorsal views (*f*). Particle paths are yellow lines and show actual locations of particles through time with thickness of lines equal to particle diameter. Edges of the tail chamber are indicated with dashed white line; note particle flow parallel to tail chamber in (*e*). Scale bars are 1 mm (*e*) and 500 µm (*f*).

Another unique feature of appendicularians is an external ‘house’ in which the mucous mesh filters are situated (figure 2) [21,22]. The houses and associated filters are temporary structures made of cellulose and protein [23,24] that are secreted from specialized oikoblastic epithelial cells on the appendicularian trunk and are inflated by tail beating [25,26]. House morphology—including the filters, fluid channels and orientation of the appendicularian—is taxon specific. Among the Oikopleuridae, the appendicularian is situated inside the house and within a cylindrical and flexible tail chamber. The beating of the tail within the chamber drives flow through the house (figure 2). On either end of the tail chamber fluid channels divide; on one end fluid is pulled from outside the house through two inlet openings and on the other fluid is pushed onto two food concentrating filters and out of the house (figure 2). Suspended particles adhere to the food concentrating filters by tangential flow filtration [20,27,28]. Following initial adhesion, particles serially detach and reattach to move toward the mouth (electronic supplementary material, video S1) [20]. This pattern corresponds with a pattern of tail beating punctuated by periodic arrest, but the fluid flow within the tail chamber has yet to be described.

Appendicularians are abundant and often dominant members of the mesozooplankton community [29–32]. They feed on marine micro-, nano- and pico-plankton, with high -predator-to-prey ratios and the capacity to concentrate particles up to 1000 times the density of surrounding suspended particles [10,16,27,33]. Yet our knowledge of how the tail beating controls fluid transport and pumping for filtration is incomplete. A description of fluid flow generated by the tail has only been shown for one appendicularian genus, *Bathochordaeus*, which focused on modelling the effects of muscular activation [18]. Unlike *Oikopleura* species, *Bathochordaeus* tail beating is not bound within a tail chamber, and how the confines of a cylindrical tail chamber control fluid flow is unknown. Furthermore, how tail beating is affected by the ranging physical conditions appendicularians experience in nature is limited. For example, tail beat frequency has been shown to increase with temperature in other planktonic organisms [34,35]. But little is known about how temperature influences the kinematics of the appendicularian tail as it generates flow for filtration of food particles.

Here, we investigate the kinematics of the tail and hydrodynamics through the tail chamber in *O. dioica.* We describe the morphology of the tail chamber and how attachment points seal and pressurize the house. Finally, we test the effects of temperature on flow rates and suggest biomimetic applications for this unique pump.

## 2. Methods

Most visualizations and experiments of the appendicularian *Oikopleura dioica* feeding in the house were conducted at the Sars Centre for Marine Molecular Biology, Bergen, Norway, using laboratory-cultured individuals [36] in December of 2015. Additional visualizations of *O. dioica* from natural populations were obtained at Friday Harbor Laboratories, San Juan Island, WA (free-swimming individual) and at the Oregon Institute of Marine Biology, Charleston, OR. We used high-resolution, high-speed micro-videography to visualize the tail kinematics of appendicularians and particle movement while they were actively pumping within the mucous house or immediately after abandoning the house. The set-up for visualizations followed that described by Gemmell *et al*. [37]. Images were recorded using an Edgertronic high-speed camera (1280 × 1024-pixel resolution, 500–1000 frames s^−1^) with brightfield illumination from a fibre optic light source, or a Photron FastCam Mini Ux100 (1024 × 1024, 125–1000 frames s^−1^) with darkfield illumination from a tilting mirror base. The filming vessel was positioned on a manually adjustable stage between the light source and the camera. A long working-distance microscope objective (4× or 10×) was mounted to an adjustable-height optics clamp positioned between the filming vessel and the camera. Observations were also made using a Leica Z6 Apo microscope.

### 2.1. Kinematics and hydrodynamics

Individuals (5 or 6 days old, approx. 600 µm trunk length [36]) were filmed while actively feeding in 50 ml glass cuvettes as described above. To better aid in visualizing the tail and its interaction with the tail chamber, animals in newly formed mucous houses were placed in a dilute milk (Tinemelk low-fat milk, 1.2% fat) bath (approx. 1 : 10 000 milk: seawater) to filter for approximately 3 min and then placed in clean seawater for an additional 3 min prior to videography to remove any non-adhered particles. Deposition of the milk fat particles (less than 1 µm in size) facilitated visualizations of the filter fibres. Subsequent velocity and morphometric measurements were performed with image stacks using the open-source software ImageJ.

Fluid motion along the tail while actively pumping within a mucous house was quantified using two-dimensional micro particle image velocimetry (µPIV) [37]. Flow tracers consisted of live, unicellular microalgae. *Rhinomonas reticulata* was used for observations at low magnifications (4×) and *Isochrysis galbana* was used for observations at high magnifications (40×). Both live particle types are small enough to pass through the inlet filter and within the size range of particles ingested by *O. dioica* (0.2–10 µm) [22,33,36,38–40]. Likewise, fluid motion along the tail for a free-swimming individual was quantified using µPIV and manual particle tracking from high-speed videos using microalgae as the flow tracer. The velocities of particles were determined from sequential images analysed using a cross-correlation algorithm (LaVision software). Image pairs were analysed with shifting overlapping interrogation windows of decreasing size of 64 × 64 pixels to 32 × 32 pixels or 32 × 32 pixels to 16 × 16 pixels to produce velocity vectors and vorticity. Streakline images (figure 2*e,f*) were created in ImageJ to show the flow of particles through the house by tracing their movement from spatially registered frames. Images were made from a maximum-projection of sequences or by compositing a single original frame with a computed image of just the moving particles. Computed images of moving particles, or streaklines, were made by first subtracting a median-filtered copy of each frame, then computing a frame-to-frame difference, and maximum projecting subsets of frames.

### 2.2. Temperature experiments

To determine if small (day 1, approx. 140 µm trunk length, [36]) *O. dioica* swimming kinematics and filtration capacity shift with changes in temperature, three temperature treatments were used: 5°C, 15°C and 25°C. These temperatures were selected because they fall within the natural range commonly encountered by *O. dioica* [41–43]. It should be noted that temperature cannot be completely decoupled from viscosity as these temperatures have associated changes in seawater kinematic viscosity (*ν*_5_ = 1.52 × 10^−6^ m^2^ s^−1^, *ν*_15_ = 1.13 × 10^−6^ m^2^ s^−1^, *ν*_25_ = 0.89 × 10^−6^ m^2^ s^−1^ [44]). Reynolds numbers (*Re*) in the tail chamber were calculated for each temperature treatment by

$$Re=\frac{UL}{v},$$

where the velocity (U) is derived from particle speed and length scale (L) is the diameter of the tail chamber. The tail chamber diameter was determined from a radius equal to tail wave amplitude (*A*, figure 2).

Filming was performed within a 50 ml glass cuvette, as above, and animals were allowed to acclimatize for 30 min before filming. Videos of tail kinematics and particles entering the inlet filter were recorded as described above and converted to image stacks in QuickTime Pro. Tail beat kinematics and particle tracking to estimate inlet flow speeds were subsequently measured in ImageJ. Flow speeds were determined by manual particle tracking every 10 frames (0.02 s). An average of five particle speeds as well as the maximum speed were recorded for all individuals. Likewise, five tail beats were recorded per individual, with a single tail beat measured as one complete wave cycle [19].

Tail kinematics and morphological characters were measured for all animals in experiments where their tail orientation allowed. Videos from 19, 16 and 15 individuals were analysed from the 5°C, 15°C and 25°C treatments for tail beat frequency and inlet particle speeds. However, positioning did not allow morphological measurements including amplitude, wavelength, tail width and trunk length to be taken from all videos, so a reduced sample size was used (*n* = 13, 9 and 4 for 5°C, 15°C and 25°C, respectively). Trunk length, tail width, wavelength and wave amplitude were averaged from five frames per animal, with each frame taken at the maximum angle of the tail–trunk junction. Amplitude was measured as the maximum height from a line originating at the tail–trunk junction and bisecting the tail at the inflection point [18,19,45]. Two amplitudes and one wavelength, tail width and trunk length measurement were taken for each frame. Tail wave amplitudes were normalized to wavelength to account for individual variation in size. Amplitude measurements per individual were an average of two measurements (i.e. amplitude of the wave crest and trough) for each of five frames. Statistical comparisons across temperature treatments of wave amplitude, tail beat frequency and particle speed were carried out using RStudio (v. 2023.03.1 + 446). Other measurements served as constants in calculations for *Re* and volume flow rates.

Finally, we calculated volume flow rates (Q) through the tail chamber for each temperature treatment using two methods: one based on particle speed as measured by particle tracking and the second using tail kinematics. We determined flow rates using the average inlet particle speed multiplied by the cross-sectional area of the tail chamber. We assumed the tail chamber was a cylinder (with cross-sectional area a circle) with radius equal to the tail wave amplitude (A). Tail kinematics flow rates were generated from tail width (W), amplitude (A) and wavelength ( λ)  and tail beat frequency (f) as has been done for other appendicularian species [16,27,46]. We used a model from Katija *et al*. [16], which was modified from Morris & Deibel [27], where the filtration volume equals a product of the wavelength, frequency and total area bound by the tail in the tail chamber (*A*_total_). The biomechanical model from Katija *et al*. [16] assumes that the total area delineated by the parabolic shape of the tail in the tail chamber, or *A*_total_ = 2/3*Aλ*. Thus, the volume flow rate is

$$Q=\frac{2}{3}W\cdot f\cdot A\lambda .$$

Although we use the term flow rate, it should be noted that other terms are used in the literature (e.g. pumping rate, clearance rate, filtration rate) and are interchangeable when derived from similar models [16,46,47]. We assumed that all time in the house was spent actively pumping and feeding, although other authors have accounted for the proportion of time feeding only [46]. We observed house-bound *O. dioica* to be almost constantly feeding, except for intermittent tail arrests (see Discussion).

## 3. Results

### 3.1. Kinematics and fluid dynamics of tail beating in *Oikopleura dioica*

**Figure 3.**
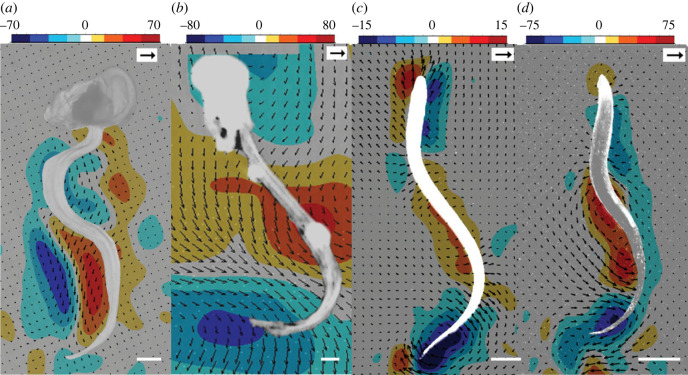
Shape of beating tail and resultant flow fields generated by a filtering appendicularian inside a mucous house (*a*). Note that flow fields are predominantly parallel to the tail. This contrasts with free-swimming *O. dioica* (*b*) and other undulatory swimmers ((*c*) larval lamprey and (*d*) chaetognath) where flow is perpendicular to the tail bend. Scale bars 100 µm (*a,b*), 1 cm (*c*) and 1 mm (*d*). Vorticity (s^−1^) scales are shown for each panel and black arrows indicate vector magnitude (10 mm s^−1^ (*a*), 20 mm s^−1^ (*b*), 80 mm s^−1^ (*c*), and 100 mm s^−1^ (*d*)).

Spatio-temporal observations of fluid motion during feeding in *O. dioica* from µPIV revealed that flow into the appendicularian house is generated by the undulating movements of the tail within the tail chamber (figure 3), and the resulting flow pattern is established by the fit of the tail within the chamber. Unlike a vortex-based pattern of fluid motion in anguilliform swimmers, where opposing and paired vortices result from undulatory movements [48–50], we observed velocity fields from immediately paired vortices moving down the tail chamber, suggesting the tail is acting more like a pump (compare figure 3 panels). Discrete packets of fluid were transported by each tail undulation with the hydrodynamics confined by the walls of the tail chamber.

Free-swimming appendicularians are particularly challenging to capture in a narrow focal plane during videography as they translate rapidly. Observations of free-swimming *O. dioica* were obtained from µPIV, opportunistically, from a single individual just after it abandoned its house. Tail kinematics and fluid mechanics in this free-swimming appendicularian qualitatively matched visualizations from other undulatory swimmers (compare figure 3 panels) and differed from the filtering appendicularian (figure 3*a*). Specifically, the fluid motion was predominantly perpendicular to the tail in free-swimming appendicularians whereas in house-bound appendicularians, the fluid moves parallel to the tail allowing for effective pumping. Despite vortices being oriented differently, vorticity maxima are similar near the tail in both free-swimming and house-bound appendicularians (figure 3*a,b*). The vorticity maxima of swimming chaetognaths are also like those observed in *O. dioica*, although chaetognaths are larger and generate faster fluid speeds (compare figure 3*a,b* with *d*).

**Figure 4.**
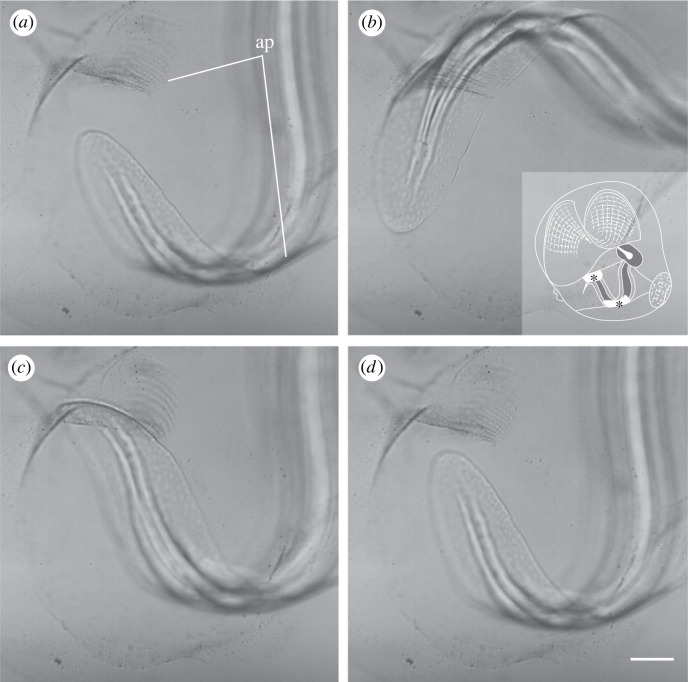
Fit of tail within the tail chamber and two attachment pads reduce backward flow in the tail chamber and increase pressure within the appendicularian house. Two attachment pads are on opposite sides of the tail chamber (ap, (*a*)). Frames from electronic supplementary material, video S3 (*a–d*) show attachment pad position, morphology and Velcro-like connection to tail. Insert in (*b*) shows position of attachment pads within the tail chamber (black asterisks). Frames are from 1 s increments; scale bar is 100 µm.

**Figure 5.**
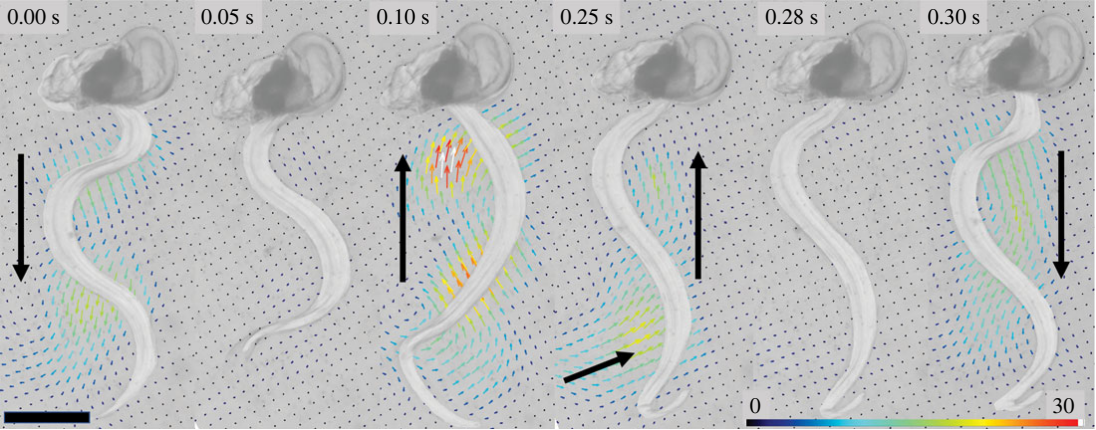
The leak-proof seal created by contact between the tail and the attachment pads on the tail chamber wall is shown with flow fields from a series of video frames. During active pumping the tail is in contact with the attachment pads, and flow is unidirectional and parallel to the tail (0.00 s, 0.30 s). Immediately before a tail arrest, the tail/attachment pad contact remains but the tail is not pumping and particle motion ceases completely (0.05 s). Once the contact between the tail and tail chamber walls is removed, there is a fast reversal in flow (0.10 s). This flow reversal remains (0.25 s) until contact between the tail and tail chamber is resumed, where particle motion ceases again (0.28 s) just before resumption of active pumping (0.30 s). Note that the initial fast flow reversal emphasizes the build-up of pressure within the house (0.10 s) which was created by the tail actively pumping. Black scale bar is 0.5 mm and colour bar indicates flow speeds in mm s^−1^.

Our micro-videography confirms that the beating tail in *O. dioica* acts as a positive displacement peristaltic pump when filtering (electronic supplementary material, video S2). Within the tail chamber, tail beating is sinusoidal and motion emanates from the tail/trunk junction with a travelling wave that moves through the end of the tail. The flexible tail chamber is sized to the appendicularian tail. The edges of the tail contact the sides of the tail chamber walls, which flex as tail beats pass (electronic supplementary material, video S4). Where the tail contacts the top and bottom of the tail chamber there are inconspicuous attachment ‘pads’ (figure 4). These pads are flexible and have distinct ridges such that the attachment to the tail itself is Velcro-like and peels away antero-posteriorly at detachment (figure 4; electronic supplementary material, video S3). This connection between tail, tail chamber walls and attachment pads apparently creates a seal that pressurizes the house, allowing fluid to be transported to the food concentrating filters with limited backward flow (electronic supplementary material, video S2). When the tail stops beating but is still attached to the pads, particle motion ceases (figure 5). Once the tail detaches from the pads during tail arrest, the house becomes depressurized and the particles reverse direction (figure 5*,* 0.10 s). The high flow magnitude observed at this reversal suggests that substantial pressure builds while the tail is pumping before the arrest.

### 3.2. Effects of temperature on tail beating kinematics in filtering *O. dioica*

To test if tail beat kinematics shift with increasing temperature, we analysed tail beat frequency, amplitude and inlet particle speeds for individuals in seawater at three temperature treatments. Individuals in experiments were 1 day old with trunk length 140 ± 0.1 µm, wavelength 0.27 ± 0.04 mm and tail width 0.064 ± 0.01 mm.

Distributions of tail wave amplitude met the assumptions of normality (Shapiro–Wilk *W* = 0.96, *p* = 0.4) and homogeneity (Bartlett's *K*^2^ = 5.57, d.f. = 2, *p* = 0.06), and treatment effects were tested using a parametric one-way ANOVA. Log-transformed tail beat frequency data met the assumption of equal variance using a Fligner–Killeen test ($\chi_{2}^{2}=0.22$, *p* = 0.9), but were still not normally distributed (*W* = 0.91, *p* = 0.001). Similarly, log-transformed particle speed data were normally distributed (*W* = 0.99, *p* = 0.35), but did not have equal variance ($\chi_{2}^{2}=10.3$, *p* < 0.001). We proceeded to test the effects of temperature using a non-parametric Kruskal–Wallis rank sum test for both log-transformed data distributions.

Temperature had a significant effect on tail beat frequency, which increased by 366% from 5 to 25°C ($\chi_{2}^{2}=42.6$, *p* < 0.0001), with 2.96 ± 0.44 beats s^−1^ at 5°C, 6.49 ± 0.90 beats s^−1^ at 15°C and 10.82 ± 1.23 beats s^−1^ at 25°C. Likewise, inlet particle speeds increased with increasing temperature ($\chi_{2}^{2}=35.95$, *p* < 0.0001), with average speeds 0.469 ± 0.186, 0.682 ± 0.186 and 0.740 ± 0.315 mm s^−1^ in the 5°C, 15°C and 25°C treatments, respectively. However, temperature did not have a significant effect on wave amplitude (*F*_1, 24_ = 3.94, *p* = 0.06), which was 0.08 ± 0.001, 0.09 ± 0.001 and 0.09 ± 0.001 mm for the three treatments. We calculated *Re* for each temperature treatment and found all treatments to have *Re* < 1 (table 1).

**Table 1.** Estimated Reynolds numbers (*Re*) for each experimental temperature treatment and associated seawater kinematic viscosity (*v*). Reynolds numbers calculated with length scale (*L*) being the diameter of the tail chamber and velocity values (*U*) estimated from an average of maximum inlet particle speeds for individuals in each treatment.

treatment	viscosity *v* (m^2^ s^−1^)	inlet particle speed *U* (mm s^−1^)	tail chamber diameter *L* (mm)	*Re*
5°C	1.52 × 10^−6^	0.86 ± 0.32	0.18	0.10
15°C	1.13 × 10^−6^	1.43 ± 0.33	0.18	0.23
25°C	0.89 × 10^−6^	1.73 ± 0.59	0.18	0.35

**Figure 6.**
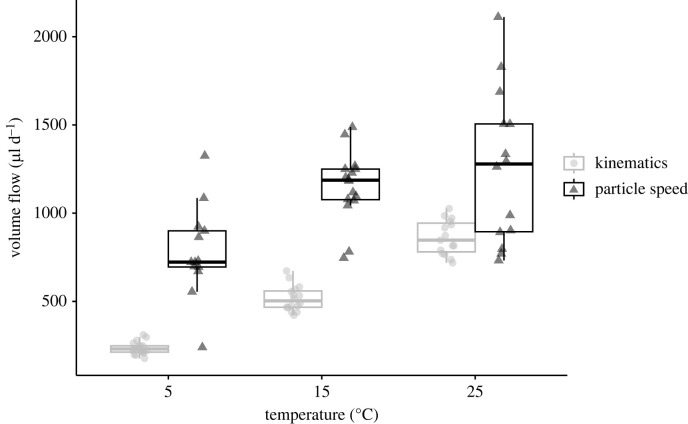
Volume flow rate through the tail chamber increases with temperature in *O. dioica*. Shown are volume flow rate estimates based on tail kinematics (grey circles) and inlet particle speed (black triangles). Note that estimates based on particle speed are consistently higher than those based on tail kinematics.

The results of our volume flow rate calculations using particle speed were higher than those using tail kinematics (figure 6). We calculated volume flow per individual per day to be 235.36 ± 35.09 µl d^−1^ at 5°C, 516.82 ± 71.84 µl d^−1^ at 15°C and 861.47 ± 97.81 µl d^−1^ at 25°C using tail kinematics. On the other hand, volume flow rates were 779 ± 260 µl d^−1^ at 5°C, 1153 ± 195 µl d^−1^ at 15°C and 1258 ± 432 µl d^−1^ at 25°C using average inlet particle speed measurements.

## 4. Discussion

When *O. dioica* are filtering within their mucous house, they display distinct kinematics and fluid motion compared with free-swimming appendicularians and from other species that exhibit undulatory swimming. In free-swimming appendicularians and small undulatory swimmers that are free from confining walls, flow is directed perpendicular to the tail. In most undulatory swimmers, body bending generates pressure fields that serve to generate forward thrust for swimming [49]. However, when appendicularians are actively filtering, the undulating tail serves a different purpose: it drives a pump that moves fluid parallel to the tail within the confines of a tail chamber while the appendicularian remains relatively static. This appendicularian pump combines aspects of several technical pump designs. It has been described as both peristaltic pump-like [17,51] and as a natural example of a stationary, dynamic fluid pump [18]. Direct flow visualizations reveal that fluid flow occurs by distinct compartments as in a positive displacement or peristaltic pump, but unlike other peristaltic pumps, pumping is driven by dynamic tail movement [2]. Among pelagic tunicates, this fluid pumping is unique to appendicularians and the fluid flow we describe in *O. dioica* is probably unique relative to other types of animals that employ undulatory body kinematics due to the appendicularian tail situated within a tail chamber.

The fluid flow is qualitatively similar between free-swimming appendicularians and other undulatory swimmers. However, undulatory swimmers vary widely in size and therefore operate at different fluid dynamical regimes. In the comparisons we make between *O. dioica* and two small undulatory swimmers (figure 3), the *Re* vary according to size. For example, the fluid motion generated by the free-swimming appendicularian (approx. 0.1 cm) is dominated by viscous forces (lower *Re*) and a laminar flow regime compared with the turbulent flow regime of the larger larval lamprey (higher *Re*, approx. 10 cm). Despite the variation in *Re*, the free swimmers that are not bound by tail chamber walls generate similar flow. Therefore, it is evident that the unique fluid pumping in filtering appendicularians arises because the tail is confined by a tail chamber.

**Figure 7.**
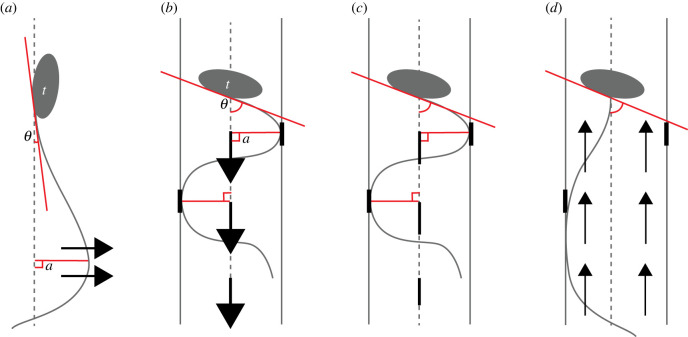
Hypothetical diagrams comparing appendicularian tail movement while free-swimming (*a*), actively pumping (*b*), immediately upon stopping (*c*), and during tail arrest (*d*). Trunk (*t*), trunk angle (*θ*) and wave amplitude (*a*) are indicated in (*a,b*); note larger trunk angle and wave amplitude when pumping. Trunk angle is shown relative to the body's midline [42,47]. Fluid flow is shown with black arrows; perpendicular to the tail when free-swimming and not confined to a tail chamber (*a*), parallel to tail when actively pumping in the tail chamber (*b*), fluid flow ceases when not actively pumping (*c*), and backward flow occurs during tail arrest (*d*). The tail chamber is sealed when the tail is in contact with attachment pads (black lines on tail chamber) (*b,c*), and backward flow is observed when this contact is removed (*d*).

Previous researchers have questioned the purpose of the structures we describe here as attachment pads on the tail chamber walls in *O. dioica*. These striated regions have been called antifriction pads [52], pockets [53], or even vents [27] in other *Oikopleura* species. Our observations of the attachment pads within the tail chamber suggest that they are subtle yet critical components for effective pumping. These apparently sealing attachment pads and precision fit of the tail within the tail chamber suggest a design that seals the tail chamber, thereby limiting backward flow of fluid while pumping (figures 5 and 7). This supports suggestions by Morris & Deibel [27] that the tail and tail chamber create a leak-proof seal, and we propose that the attachment pads are a necessary component of the seal.

*Oikopleura dioica* creates pulses of flow by coupling pumping with intermittent arrests, which facilitates captured particle movement for ingestion [20,54]. When the tail is removed from the pads during arrest the house becomes de-pressurized and water flows backwards (figure 5*,* 0.10 s). These cycles of pressurization and de-pressurization are critical for filtration. Briefly, tail beating inflates the food concentrating filters within the house, increasing the length of the elastic, mucous mesh fibres. At the same time, flow resulting from tail beating concentrates food particles on those filters [20]. Tail arrest relaxes the inflation and decreases the length of the mucous fibres. The subsequent tail beating rapidly drives flow to reinflate the filters. This sudden lengthening of the mucous fibres detaches particles concentrated on the mucous mesh allowing them to move and reattach toward the buccal tube (electronic supplementary material, video S1, [20]). The fluid movement in the pump-like tail chamber revealed in this study explains how sufficient build-up of pressure is generated to detach food particles.

While many animals use flagellar or ciliary beating to generate flow for feeding, analogues to the appendicularian pump are few. Sponges (phylum: Porifera) are the epitome of suspension feeders; their bodies are largely composed of specialized filtering cells, the choanocytes, whose morphology is not unlike the appendicularian: flagellar movement arises from a static body that is confined within a collar [7]. The choanocyte pump also has a peristaltic design with a choanocyte collar that similarly encloses the flagellum and close packing of many neighbouring cells [4,17,55,56]. However, the choanocyte pump has been described as ‘leaky’ [4] and the generation of flow by choanocytes is solely to concentrate particles; there is no house where a build-up or dynamic change in pressure would be beneficial (discussed above). Perhaps a better analogue to *Oikopleura's* pump is the flame-cell protonephridium, in which a blade composed of many cilia executes a flagellar beat at the head of a long duct, driving fluid across an ultrafiltration membrane. The flame-like flagellar beat within a duct is like the appendicularian tail within the chamber, yet these internal structures filter out large particles and drive flow of liquid waste from the body [57,58]. Further, it is unknown if this pumping is peristaltic as it is in appendicularians.

Changes in seawater temperature drive associated changes in kinematic viscosity. For planktonic organisms operating at low *Re*, low temperatures and resultant high fluid viscosities can lead to reduced swimming speed [34,59,60] or changes in body movement [61] or morphology [62,63]. At the same time, seawater temperature has physiological effects on planktonic organisms, and increasing temperature can lead to increased swimming speed [35] or feeding rates [64]. Our results revealed a tripling in tail beat frequency and concomitant doubling in filtration rate in *O. dioica* as temperature was increased from 5 to 25°C (figure 6). As our experiments did not decouple viscosity from temperature, our tail beat frequency measurements should not be attributed to temperature alone. Podolsky & Emlet [34] separated the effects of temperature and viscosity on swimming in sand dollar (*Dendraster excentricus*) larvae over a similar range in temperatures used here (10–22°C). They found a 40% reduction in swimming speed due to an increase in viscosity while the remaining 60% was due to temperature. We assume that the increase in tail beat frequency observed here was due to a combination of increasing metabolic response and decreasing kinematic viscosity, and further work is needed to divide these effects. In spite of shifting tail kinematics and fluid mechanics as temperature increased, tail amplitude remained constant, serving to maintain the contact with the attachment pads required to seal the tail chamber for peristaltic pumping.

Clearance rates have been measured for several appendicularians, including *in situ* observations [16,21,27,46,65] and laboratory experiments [26,52,66,67] (summarized in [68]). Previous research with *Oikopleura* species has shown clearance rates to be correlated with temperature, food concentration and trunk length [27,65–67]. The appendicularians in our study were smaller than those in previous work, so it is not surprising that our volume flow rate estimates are also smaller than those previously measured. However, if we calculate a clearance rate using our study parameters (trunk length 140 µm at 15°C) and a linear regression from laboratory incubations by Broms & Tiselius [66], we find a clearance rate of 650 µl per day. This is between our two flow rate estimates (1152 ± 195 µl using particle speed and 516 ± 72 µl using tail kinematics) and confirms that similar results can be achieved using these different methods.

Kinematic tail motion and particle speed velocities represent two distinct ways of estimating flow rate and produced different, though overlapping, values (figure 6). Tail beating is a stereotyped, invariant motion that produces a tighter range of flow rate values. Tail kinematic measurements are arguably easier to obtain than particle speeds, especially from *in situ* videography, and have been successfully obtained previously [16,27,46]. Particle speeds, on the other hand, provide a more direct measurement of the maximum fluid processing capabilities and produced higher, but more variable, values. Non-invasive kinematic measurements are particularly advantageous for fragile organisms like appendicularians but future studies should acknowledge that they may produce underestimates of the volume filtered compared with direct particle velocity measurements, because kinematic measurements are an indirect, approximate measure of the fluid flow and do not account for the nuance of fluid-structure interactions as the fluid moves through the tail chamber.

**Figure 8.**
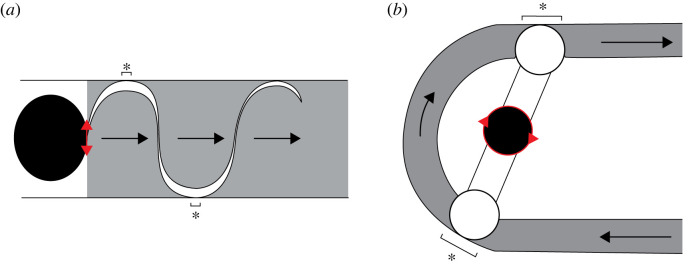
Simplified comparison of the appendicularian pump (*a*) and an example of a peristaltic pump (*b*, roller pump after [1]); grey regions indicate transported fluid and white regions show displacement chambers, resulting from contact with or compression of tube walls, which separate discrete fluid parcels. Note smaller displacement regions (indicated with asterisks) where tail contacts tail chamber in the appendicularian compared with those from tubing, which is compressed by rollers (white circles) in (*b*). Red arrowheads indicate direction of movement and location of actuator; black arrows show direction of fluid flow.

Appendicularians are ubiquitous filter feeders in oceanic mesozooplankton communities. Their ability to filter and concentrate small, suspended particles comes from their unique hydrodynamic pumping strategy. Suggestions for bioinspired underwater vehicle and robotic design have focused on the kinematics and hydrodynamics of swimming in fish and invertebrates [13–15,55,69–71]. The range of mechanisms that suspension feeding metazoans employ to separate particles from a fluid suspension is another fertile ground for biomimetic applications [28]. We suggest that the distinctive features of the appendicularian pump could inspire technical pump designs. A potential advantage of this natural pumping design includes an internal actuator that drives flow, rather than external compression, which can fatigue rollers or flexible tubing and membranes. While tail bends result from muscular contraction down its length in appendicularians, technical design could use flexible materials with desired properties to achieve fluid transport for different *Re* (e.g. [18,56,72]). In positive displacement pumps (e.g. peristaltic pumps), flow is pulsatile due to displacement chambers necessary for the transport of fluid and dampening those pulses could improve pump design [1]. With an internal flexible blade transporting fluid without compression, displacement regions (and resulting pulsatile flow) would be reduced, resulting in a more energetically efficient pump (figure 8).

### 4.1. Concluding remarks

The appendicularian house is a unique and elaborate feeding structure with equally unique fluid dynamics. The ability to filter a large volume of water, thereby concentrating food particles, depends on a pumping mechanism capable of maintaining enough pressure to keep the mucous house inflated yet also capable of rapid deflation and inflation cycles to dislodge trapped particles. Here, we describe the interaction of the appendicularian tail with its chamber as a pump that enables flow and filtration in the house. We suggest that this effective but simple peristaltic pump design could be another example of bioinspired design from a pelagic tunicate.

## Data Availability

Raw data have been uploaded to the Biological and Chemical Oceanography Data Management Office (BCO-DMO) and are available from the following links: https://www.bco-dmo.org/dataset/897617; https://www.bco-dmo.org/dataset/897682; https://www.bco-dmo.org/dataset/897825. Supplementary material is available online [73].
